# Non-invasive perfusion territory quantification and time-resolved angiography by arterial spin labeling in a patient with a large right-hemispheric AVM: case report

**DOI:** 10.1007/s00415-022-11065-3

**Published:** 2022-03-12

**Authors:** Moritz R. Hernandez Petzsche, Miriam Reichert, Gabriel Hoffmann, Hans Liebl, Michael Helle, Makoto Obara, Maria T. Berndt, Claus Zimmer, Tobias Boeckh-Behrens, Stephan Kaczmarz, Nico Sollmann

**Affiliations:** 1 Department of Diagnostic and Interventional Neuroradiology, School of Medicine, Klinikum rechts der Isar, Technical University of Munich, Ismaninger Straße 22, 81675 Munich, Germany; 2 TUM-Neuroimaging Center, Klinikum rechts der Isar, Technical University of Munich, Munich, Germany; 3 Department of Radiology, Neuroradiology and Minimal-Invasive Therapy, Klinikum Bogenhausen, Munich, Germany; 4 Philips Research, Hamburg, Germany; 5 Philips Electronics Japan Healthcare, Tokyo, Japan; 6 Philips Healthcare, Hamburg, Germany; 7 Department of Diagnostic and Interventional Radiology, University Hospital Ulm, Ulm, Germany

Dear Sirs,

Brain arterio-venous malformations (AVMs) are high-flow cerebral shunt systems that may cause severe intracranial bleeding [1]. Often, AVMs are incidentally found on brain imaging performed for a different purpose. Brain AVMs have an annual bleeding rate of 2–3% [2]. AVMs that are large or that have previously bled show a higher risk of bleeding [2, 3].

Computed tomography (CT) angiography or time-of-flight (TOF) magnetic resonance angiography (MRA) are suited methods for primary diagnosis of brain AVMs. However, to reliably localize all vascular feeders and their architecture, digital subtraction angiography (DSA) is frequently required. DSA is also more sensitive in localizing venous drainage, an important criterion for estimating surgical risk based on the Spetzler–Martin grading system [4]. Furthermore, DSA allows for evaluation of altered flow dynamics and changes of vascular territories due to AVMs and, therefore, is indispensable for planning a surgical or endovascular therapeutic intervention. MRI-based alternatives are sought after due to the invasive nature and radiation exposure of DSA, especially for non-emergent cases and for patient follow-up examinations.

In patients with large brain AVMs, additional to the risk of bleeding, a blood steal phenomenon for the surrounding brain tissue is likely, possibly leading to border zone shifts [5, 6]. Conventional MRI methods including dynamic susceptibility contrast perfusion imaging have been used to localize watershed areas of border zones between vascular territories and to quantify their spatial shifts, primarily among patients with internal carotid artery (ICA) stenosis [7, 8]. A non-invasive alternative to dynamic susceptibility contrast perfusion imaging is super-selective arterial spin labeling (ssASL) [9]. This technique allows delineation of perfusion changes for individual arterial territories without the use of a contrast agent [9]. More recently, a super-selective 4D ASL-based MRA technique (4D-sPACK) has been introduced, which represents a non-contrast-enhanced, time resolved, dynamic MRA approach for selective visualization of arterial vessels [10, 11]. The method demonstrated high diagnostic consensus with DSA in identifying arterial feeders and deep venous drainage of brain AVMs [10].

In this case report, we seek to confirm high imaging concordance between DSA and 4D-sPACK for an illustrative case of a patient with a large right-hemispheric AVM. Furthermore, we aim to demonstrate that ssASL-based perfusion maps allow delineation and volumetric quantification of individual brain vascular territories. We herein introduce a novel approach for semi-automated segmentation of ssASL-based perfusion maps, yielding masks of individual perfusion territories that allow the quantification of shifts of perfusion territories.

A 25-year-old man presented to the emergency department of a peripheral hospital with acute onset headache, paresthesia of the left hand, nausea and vomiting. The patient reported previous episodes of transient paresthesia of the left-hand and right-sided headaches. A non-contrast head CT and a cerebral CT angiography were performed, which showed a large space-occupying lesion at the level of the right basal ganglia with surrounding abnormal vascular structures. No signs of bleeding were found. The patient was transferred to our hospital for further diagnostic work-up and treatment. Upon arrival, the patient did not show any focal neurological deficits.

**Fig. 1:**
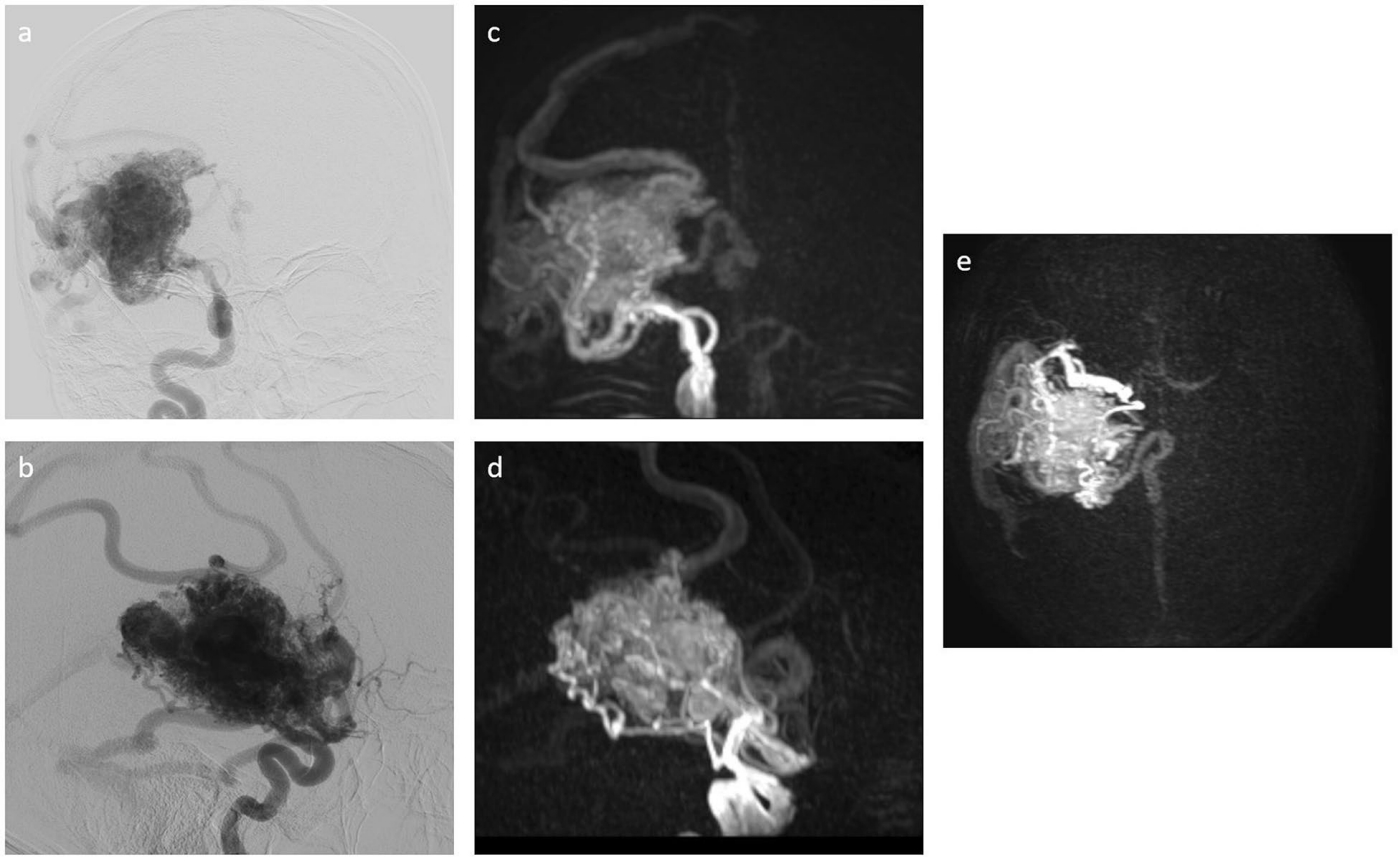
Intracranial vessel imaging—branches of the right internal carotid artery (ICA). Digital subtraction angiography (DSA) after contrast agent administration into the right ICA (**a**, **b**) and super-selective 4D arterial spin labeling (ASL)-based magnetic resonance angiography (MRA; 4D-sPACK) after automated labeling of the right ICA (**c**–**e**). Coronal views (upper row) and sagittal views (lower row) are depicted for the comparison of imaging between techniques, supplemented by an axial view for 4D-sPACK (**e**). In intermodal comparison, both techniques show a high degree of arterio-venous shunting. For 4D-sPACK, imaging with a post-label delay of 2000 ms is used

**Fig. 2:**
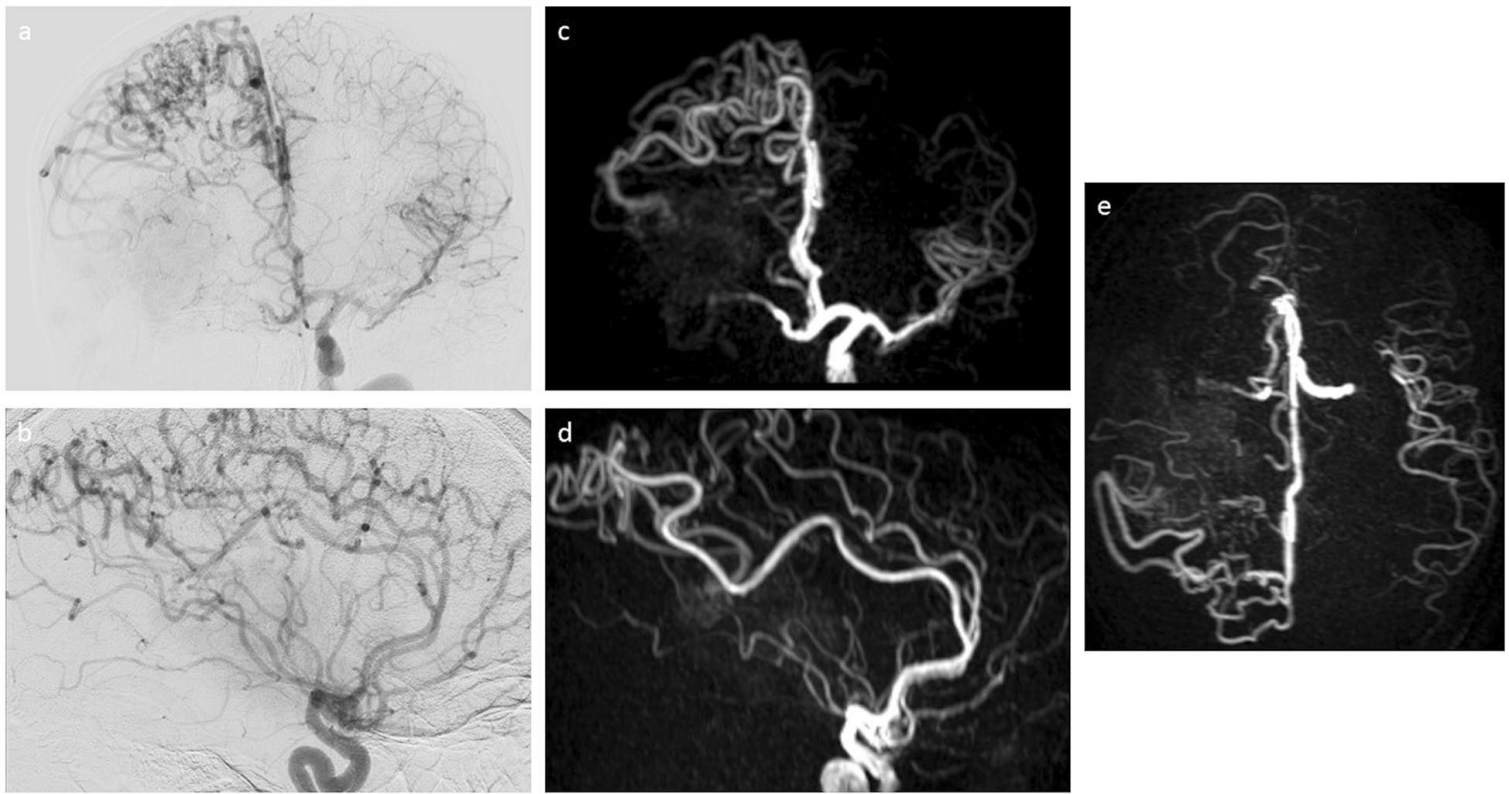
Intracranial vessel imaging—branches of the left internal carotid artery (ICA). Digital subtraction angiography (DSA) after contrast agent administration into the left ICA (**a**, **b**) and super-selective 4D arterial spin labeling (ASL)-based magnetic resonance angiography (MRA; 4D-sPACK) after automated labeling of the left ICA (**c**–**e**). Coronal views (upper row) and sagittal views (lower row) are depicted for the comparison of imaging between techniques, supplemented by an axial view for 4D-sPACK (**e**). Imaging of the intracranial branches of the left ICA shows enlarged arteries stemming from the anterior cerebral arteries and running along the superior surface of the right hemisphere. Small amounts of contrast medium in the DSA and high signal in the 4D-sPACK sequence are depicted within the arterio-venous malformation (AVM), suggesting an AVM-related feeder from the anterior cerebral arteries. For 4D-sPACK, imaging with a post-label delay of 2000 ms is used

For therapeutic planning, bi-planar DSA (Azurion; Philips Healthcare, Best, The Netherlands) was performed. Upon injection of an iodine-containing contrast agent into the right ICA, a brain AVM with a nidus of 4.5 × 6 × 8 cm was visualized (Figs. 1 and 2).

Arterial feeders were identified, especially stemming from the right middle cerebral artery, but to a lesser extent also from the right anterior cerebral artery and posterior cerebral artery. The blood flow of the right ICA seemed to be limited to the marked steal of the AVM, contributing very little to the parenchymal perfusion of the right hemisphere. Evaluation of the left ICA by DSA was additionally performed, revealing extensive crossflow to the right-sided brain parenchyma via the Circle of Willis (blood flow of both anterior cerebral arteries stemmed from the left ICA). Venous drainage occurred mostly via superficial veins into the dural sinuses. However, an ectatic deep cerebral drainage vein was also visualized (Figs. 1 and 2).

Scanning by MRI was performed on a 3-Tesla system (Achieva dStream, Philips Healthcare, Best, The Netherlands) using a 32-channel head coil and the Spinlab patch (Software release R5.6) to facilitate super-selective imaging. The standard protocol included a 3D fluid attenuated inversion recovery (FLAIR), TOF-MRA, diffusion-weighted, and T_2_*-weighted sequence. An extension of the standard protocol with 3D T_1_-weighted turbo field echo imaging and perfusion sequences was performed. Specifically, whole-brain pseudo-continuous ASL, ssASL, and 4D-sPACK sequences were acquired with labeling of the left and right ICA, using an automated label positioning tool based on TOF-MRA imaging at the neck level [12]. Parameters of these sequences were set in agreement with latest recommendations and as previously described [11, 13].

**Fig. 3:**
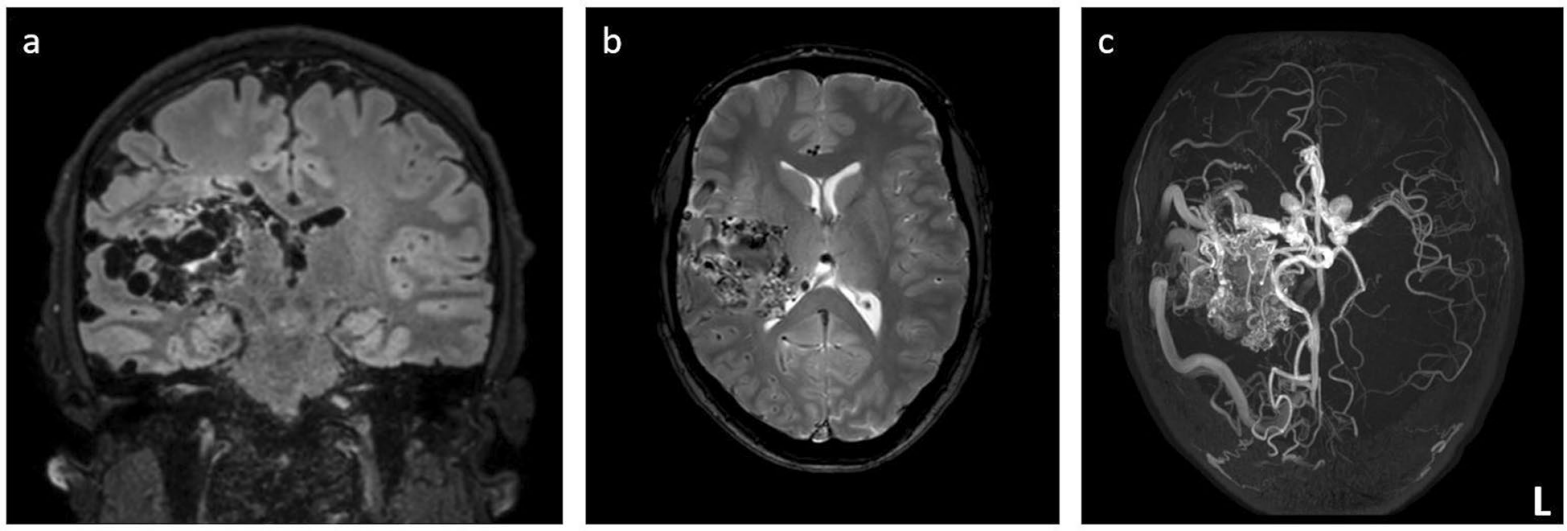
Structural magnetic resonance imaging (MRI) and conventional magnetic resonance angiography (MRA). Coronal fluid attenuated inversion recovery (FLAIR, **a**) shows a large space-occupying lesion suggestive of an arterio-venous malformation (AVM) with compression of the right lateral ventricle. According to axial T2*-weighted imaging (**b**), no evidence of bleeding was revealed. Time-of-flight (TOF)-MRA in a paraxial view (**c**) shows an increased arterial flow signal in the right hemisphere with evidence of dilated draining venous structures containing arterialized flow

FLAIR imaging revealed a space-occupying right-hemispheric lesion with densely packed central hypointense flow voids. No signs of bleeding were found on T2*-weighted imaging. The flow voids contained an arterial flow signal on TOF-MRA imaging (Fig. 3). After selective automated labeling of the right ICA using 4D-sPACK, marked shunting with little right-sided parenchymal perfusion and no signal in the right anterior cerebral artery was visualized (Figs. 1 and 2). Imaging with labeling of the left-sided ICA indicated marked extension of the vascular territory beyond the midline into the right hemisphere via dilated arteries on the superior surface of the right hemisphere, most likely stemming from the anterior cerebral arteries (Figs. 1 and 2).

**Fig. 4:**
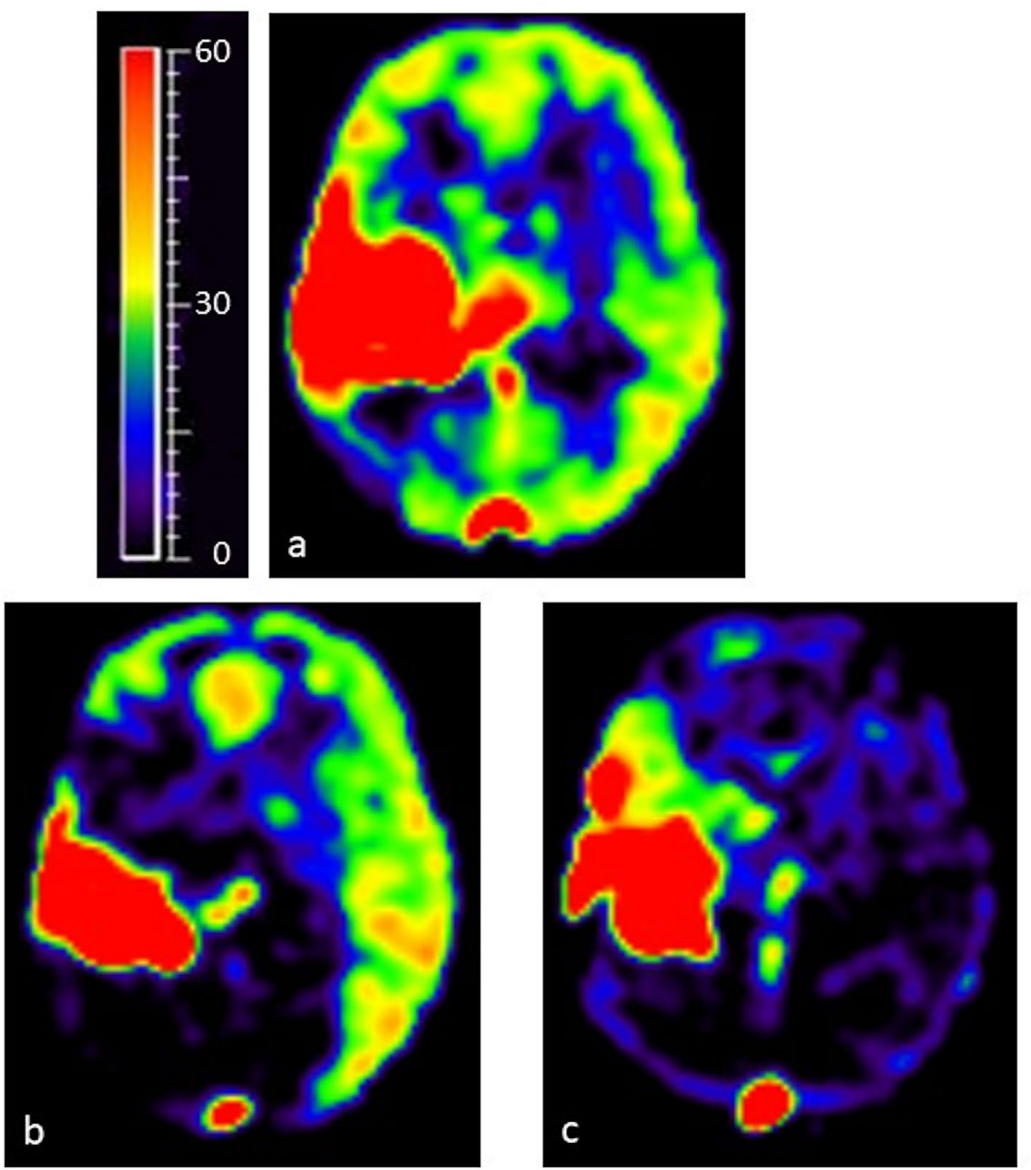
Perfusion imaging using arterial spin labeling (ASL). Cerebral blood flow is focally highly increased in the right hemisphere due to the arterio-venous malformation (AVM), as quantitatively measured by whole-brain pseudo-continuous ASL (**a**; scaling in ml/100 g/min. The perfusion territory of the left-sided internal carotid artery (ICA) as measured by super-selective ASL (ssASL) imaging is extended markedly beyond the midline into the right hemisphere (**b**). The perfusion territory of the right ICA as measured by ssASL imaging shows blood flow to the AVM with very little perfusion of the surrounding brain parenchyma of the right hemisphere (**c**)

Overall, there was a high visual concordance between DSA and 4D-sPACK regarding the localization and extent of AVM feeders and the identification of the venous drainage (Figs. 1 and 2). Furthermore, whole-brain pseudo-continuous ASL and ssASL-based perfusion maps indicated increased cerebral blood flow overlapping with the location of the AVM (Fig. 4).

**Fig. 5:**
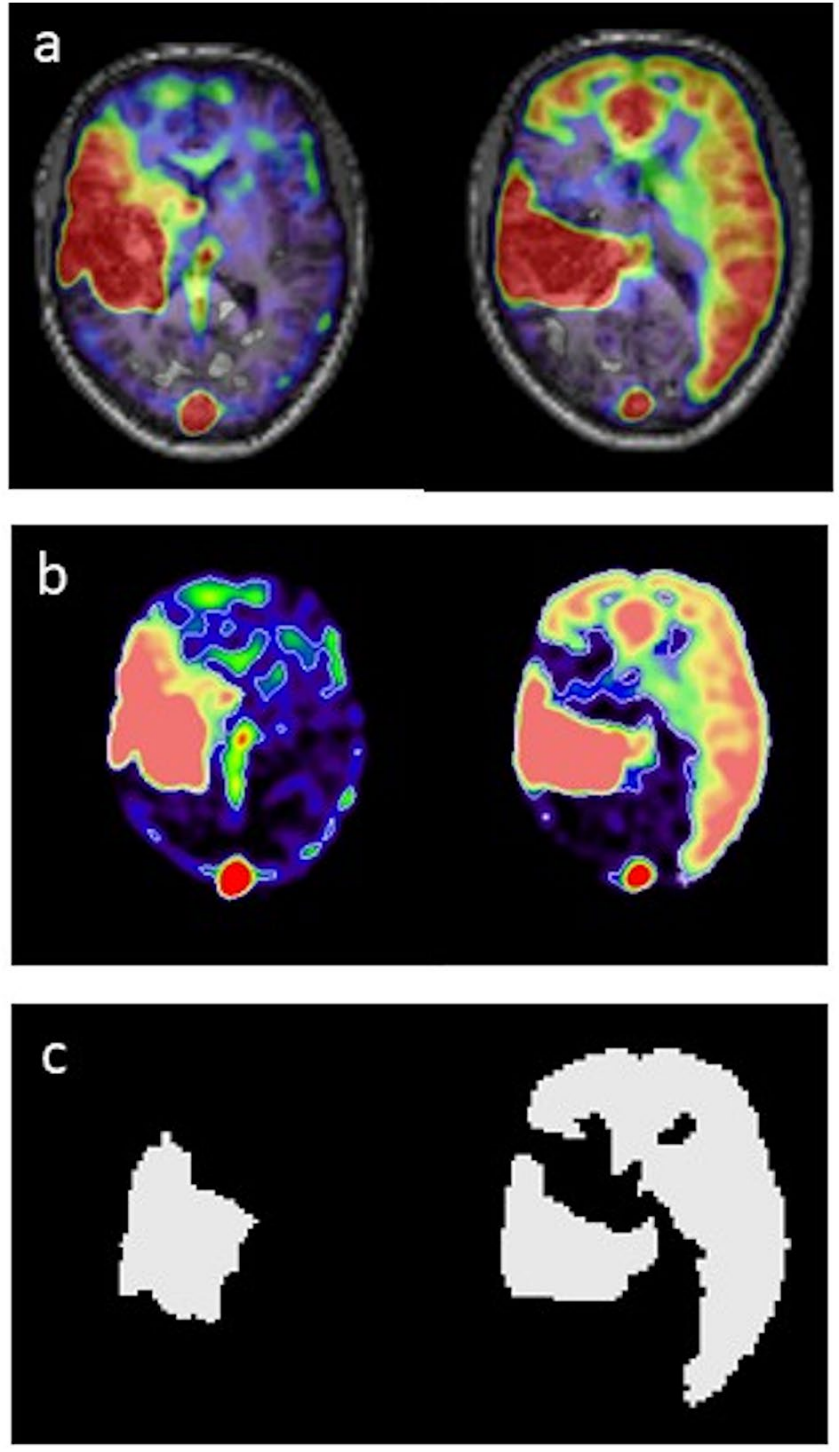
Segmentation of perfusion territories. Perfusion territories were semi-automatically segmented, using super-selective arterial spin labeling (ssASL) data with automated labeling of the respective right and left internal carotid artery (ICA). Overlays of perfusion territories co-registered with T1-weighted imaging are illustrated (**a**), and ssASL data were thresholded to derive masks of individual perfusion territories, allowing for volumetric perfusion territory quantification (**b**; color-coded perfusion masks). Binary perfusion masks after thresholding are provided in addition, showing perfused territories after thresholding as white areas (**c**)

Quantitative evaluations of perfusion territory shifts were achieved by segmenting ssASL data using a two-step approach. First, ssASL data were segmented using VINCI (“Volume Imaging in Neurological Research, Co-Registration and ROIs included”; Max Planck Institute for Metabolism Research, Cologne, Germany). The proposed semi-automated approach comprised thresholding and manual corrections to derive masks of individual vascular perfusion territories based on the supply stemming from the left and right ICA, respectively (Fig. 5). Second, quantitative analyses were performed using MATLAB 21b (MathWorks Inc., Natick, MA, USA) and SPM 12 (version 6225; www.fil.ion.ucl.ac.uk/spm; Wellcome Trust Centre, London, UK). Hemispherical shift of perfusion territories was calculated by the fractional volume shifted into the opposite hemisphere, normalized to the volume of the respective hemisphere.

In addition, hemispheric shifts of individual perfusion territory masks were compared against a vascular territory atlas of the frontal circulation [14], which was co-registered onto the patient’s ssASL data. Overlap between masks of individual territories and the atlas was calculated for both hemispheres using DICE coefficients. We calculated a shift of 71.6% from the left ICA to the right hemisphere and found a smaller overlap for the right hemisphere (DICE coefficient = 0.38) compared to the left hemisphere (DICE coefficient = 0.62).

Due to its size and supposed functionally eloquent localization, the AVM was not considered for neurosurgical resection. An endovascular treatment approach was also deemed unsafe and with low probability of success. Thus, radiotherapy was recommended as the first-line treatment.

In this case report, we show high agreement for the visualization of a large brain AVM between DSA and 4D-sPACK, and introduce an ssASL-based approach for segmentation of vascular territories with quantitative analysis. Perfusion maps derived from ssASL-based imaging may be used to create vessel-specific territorial perfusion masks, an application useful for visualizing border zones and border zone shifts in patients with cerebrovascular pathologies such as AVMs.

In the presented case of a patient suffering from a large right-hemispheric AVM, 4D-sPACK was able to correctly identify the relevant arterial feeders and venous drainage of the complex AVM with functionally eloquent location. Furthermore, there was high concordance between DSA and 4D-sPACK regarding the identification of the high degree of shunting of the right ICA, leading to it contributing little to the physiological brain perfusion. As a potential advantage over DSA, 4D-sPACK precisely depicts the pathologically changed flow dynamics, whereas arterial flow in DSA may be potentially changed by the pressure of contrast injection.

Owing to its vessel-selective approach together with the segmentation of individual perfusion territories, ssASL-based perfusion maps enable volumetric quantification of perfusion territories and border zones between vascular territories. In the presented case, a high degree of perfusion territory shift was visualized and confirmed by segmentation with quantitative verification in favor of the left ICA, due to the high degree of AVM-induced shunting regarding the right ICA. Thus, mere visual inspection of ssASL data could be markedly enhanced with the herein introduced semi-automated perfusion territory segmentation by allowing quantitative analyses on single-subject as well as group level. Accurate detection of border zone shifts using this technique is feasible and pending further study, even for pathologies where this effect could be less evident.

Knowledge of individual perfusion territories is clinically relevant in surgical planning. For instance, the watershed areas known to exist in border zones between vascular territories are particularly prone to ischemic events, especially if they are located distal of an arterial stenosis [15]. Although AVMs are not stenotic pathologies, large shunt volumes, as observed in the presented patient case, may result in a comparable intracranial hemodynamic situation. The blood steal phenomenon related to AVMs may lead to a decrease in perfusion pressure of distally located brain parenchyma, potentially leading to clinical symptoms and a need for collateralization [5, 6]. This is suggested by the high degree of border zone shift that was observed in the presented case. The right-hemispheric brain parenchyma is collateralized by dilated arteries along the superior surface of the right hemisphere, predominantly stemming from the anterior cerebral arteries and supplied by the left ICA.

Future clinical applications of ssASL and 4D-sPACK go beyond imaging of AVMs. Specifically, these MRI-based imaging techniques can be clinically used to follow up and monitor patients with Moyamoya disease without requiring repeated DSA. Furthermore, ssASL and 4D-sPACK could be used to monitor patients with in-stent stenosis after cerebral flow-diverter or stent implantation, providing information on potential consequences for brain perfusion in vessel-selective fashion. The herein proposed imaging methods may also be considered as alternatives to balloon occlusion testing, an endovascular procedure to determine safety of vessel sacrifice. All above-mentioned potential clinical applications of ssASL and 4D-sPACK may profit from the novel segmentation-based analysis approach introduced in this study, which allows objective quantification of changes in perfusion territories. In addition, automated labeling of the left and right ICA, as applied in the presented patient case, can support clinical feasibility and enhances the workflow during ssASL and 4D-sPACK acquisitions.

Vessel-selective ASL-based perfusion imaging with segmentation of vascular perfusion territories and time-resolved angiography is a promising approach to evaluate cerebrovascular pathologies like AVMs. The techniques are feasible in their clinical implementation, are non-invasive, and do not require administration of contrast media. Moreover, arterial flow dynamics can be visualized and delineated without dependence on contrast injection pressure, as it is the case for DSA. Furthermore, ssASL-based perfusion imaging with segmentation and quantitative analysis allows reconstruction of vascular territories, an application previously not achievable with other techniques. This method shows promise also for the evaluation of other neurovascular pathologies.
